# Mass spectrometric study of variation in kinin peptide profiles in nasal fluids and plasma of adult healthy individuals

**DOI:** 10.1186/s12967-022-03332-8

**Published:** 2022-03-29

**Authors:** Tanja Gangnus, Anke Bartel, Bjoern B. Burckhardt

**Affiliations:** Institute of Clinical Pharmacy and Pharmacotherapy, Heinrich Heine University Düsseldorf, Universitätsstr. 1, 40225 Düsseldorf, Germany

**Keywords:** Bradykinin, Kallikrein-kinin system, Plasma, Nasal lavage fluid, Reference levels, Nasal epithelial lining fluid, Healthy volunteers

## Abstract

**Background:**

The kallikrein-kinin system is assumed to have a multifunctional role in health and disease, but its in vivo role in humans currently remains unclear owing to the divergence of plasma kinin level data published ranging from the low picomolar to high nanomolar range, even in healthy volunteers. Moreover, existing data are often restricted on reporting levels of single kinins, thus neglecting the distinct effects of active kinins on bradykinin (BK) receptors considering diverse metabolic pathways. A well-characterized and comprehensively evaluated healthy cohort is imperative for a better understanding of the biological variability of kinin profiles to enable reliable differentiation concerning disease-specific kinin profiles.

**Methods:**

To study biological levels and variability of kinin profiles comprehensively, 28 healthy adult volunteers were enrolled. Nasal lavage fluid and plasma were sampled in customized protease inhibitor prespiked tubes using standardized protocols, proven to limit inter-day and interindividual variability significantly. Nine kinins were quantitatively assessed using validated LC–MS/MS platforms: kallidin (KD), Hyp^4^-KD, KD_1-9_, BK, Hyp^3^-BK, BK_1-8_, BK_1-7_, BK_1-5_, and BK_2-9_. Kinin concentrations in nasal epithelial lining fluid were estimated by correlation using urea.

**Results:**

Circulating plasma kinin levels were confirmed in the very low picomolar range with levels below 4.2 pM for BK and even lower levels for the other kinins. Endogenous kinin levels in nasal epithelial lining fluids were substantially higher, including median levels of 80.0 pM for KD and 139.1 pM for BK. Hydroxylated BK levels were higher than mean BK concentrations (Hyp^3^-BK/BK = 1.6), but hydroxylated KD levels were substantially lower than KD (Hyp^4^-KD/KD = 0.37). No gender-specific differences on endogenous kinin levels were found.

**Conclusions:**

This well-characterized healthy cohort enables investigation of the potential of kinins as biomarkers and would provide a valid control group to study alterations of kinin profiles in diseases, such as angioedema, sepsis, stroke, Alzheimer’s disease, and COVID-19.

## Background

**Fig. 1:**
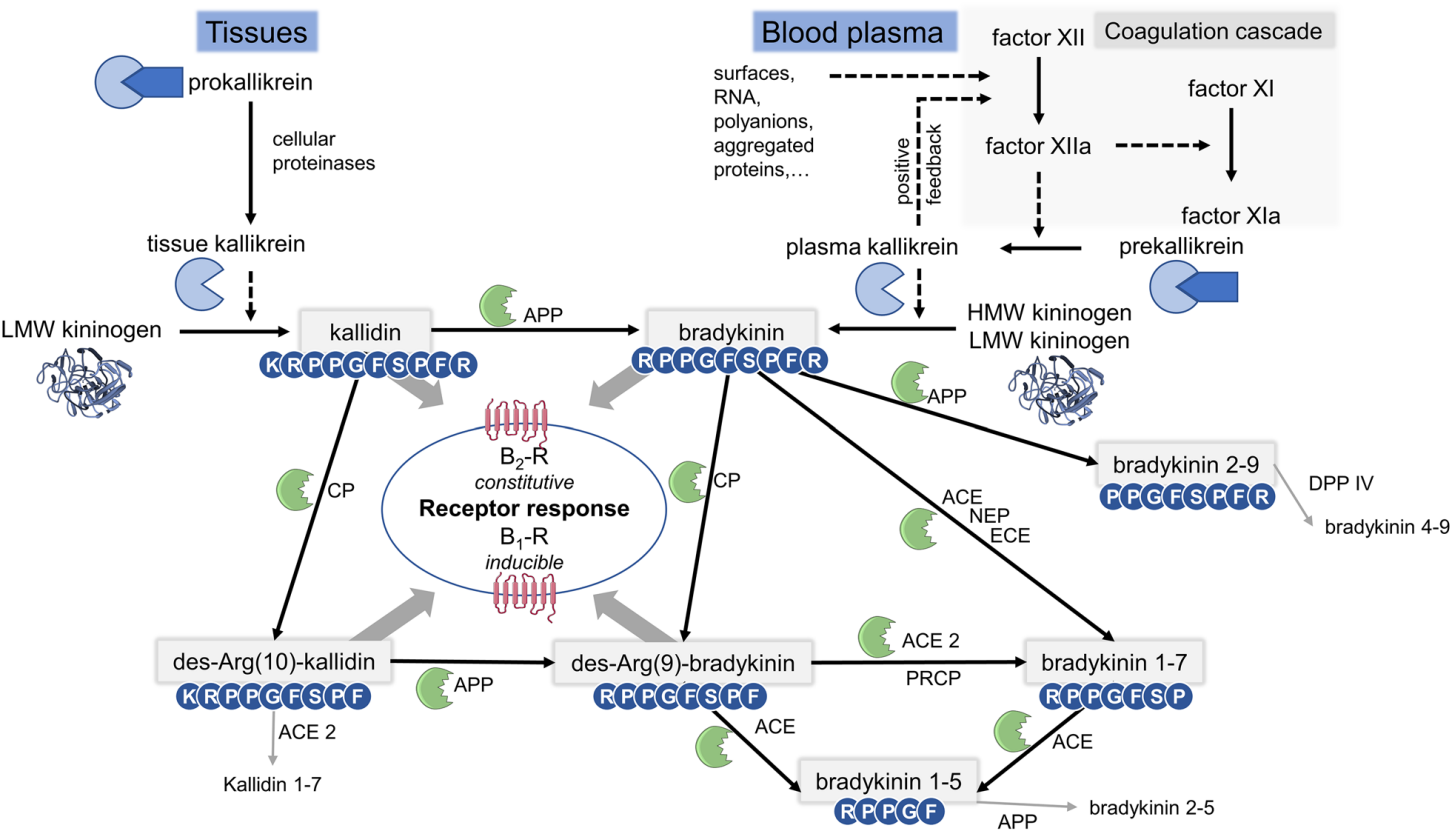
The kallikrein-kinin system. Kinins are either formed in tissues by cleavage of low molecular weight (LMW) kininogen into kallidin or in plasma via activity of plasma kallikrein and formation of bradykinin from high molecular weight (HMW) or LMW kininogen. The activity of plasma kallikrein is reciprocally amplified by activation of factor XII after contact with artificial or pathophysiological surfaces, leading to increased generation of bradykinin. While kallidin and bradykinin activate B_2_-R, their N-terminal des-Arg metabolites stimulate B_1_-R. Kinin amino acid sequences are displayed in the one-letter code. APP: aminopeptidase P, ACE: angiotensin-converting enzyme, ACE 2: angiotensin-converting enzyme 2, B_1/2_-R: bradykinin receptor type 1/2, CP: carboxypeptidase, DPP IV: dipeptidyl peptidase IV, ECE: endothelin-converting enzyme, NEP: neprilysin, PRCP: prolyl carboxypeptidase, RNA: ribonucleic acid

The kallikrein-kinin system (KKS) is a complex cascade of proteins, proteases, and active and inactive kinin peptides. The KKS is involved in physiological and pathophysiological processes and is thoroughly intertwined with the renin–angiotensin–aldosterone system (RAAS); both systems acting counterregulatory to maintain physiological hemostasis [1]. Kinins are inflammatory mediators implicated in the pathological development of cardinal signs of inflammation [2]. They exert their action by activating the G-protein coupled bradykinin (BK) receptors type 1 and 2, whereby type 1 is particularly upregulated during inflammation (Fig. 1) [3]. Despite the KKS’s proposed involvement in many diseases, such as sepsis, COVID-19, stroke, Alzheimer’s disease, and allergic reactions, hereditary angioedema currently remains the sole therapeutic application of targeting this system [2, 4–6].

Lack of data establishing kinins as biomarkers has limited their clinical use. Scientific progress regarding robust kinin quantification has lagged behind comparable systems like the RAAS despite similar advances in scientific methodologies over the past decades [7]. Determination of immunoreactive kinins does not differentiate the BK type 2 receptor agonists BK and kallidin (KD), which are released through different pathways (Fig. 1). Furthermore and particularly in plasma, BK levels varying by several orders of magnitude (low pM to high nM) have been published, hindering distinction between health and disease as well as interstudy comparisons [8–10]. This variability is attributed to the high sensitivity of kinin level results to specimen handling during the pre-analytical and analytical phase; the short half-lives of kinin peptides and artificial generation of the kinin BK in plasma via contact activation of factor XII render specimen handling technique essential (Fig. 1) [11, 12]. Moreover, existing data are often restricted on reporting levels of single kinin levels, thus neglecting the distinct effects of multiple active kinins on the two BK receptor types and diverse metabolic pathways (Fig. 1).

Recently, reliable and robust kinin determination has been investigated extensively using modern bioanalytical techniques. Improvement of the sensitivity of mass spectrometric assays established validated liquid-chromatography coupled with tandem mass spectrometry (LC–MS/MS) platforms for the comprehensive determination of active and inactive kinins in human plasma and respiratory lavage fluids [13–15]. This technique facilitated the investigation and subsequent standardization of pre-analytical variables, contributing to a substantial reduction in inter-day and interindividual variability of plasma kinin levels [16]. However, there is biological variation among healthy individuals, which might be additionally confounded by artificial kinin changes. Thus, a better understanding of kinin profiles in healthy individuals is essential for exploring and distinguishing disease-specific kinin profiles, as it has already become achievable for the RAAS [17, 18]. Understanding disease-specific kinin profiles has become a subject of focus during the COVID-19 pandemic, where increased BK_1-8_ levels secondary to decreased angiotensin-converting enzyme 2 activity after binding of severe acute respiratory syndrome coronavirus 2 (SARS-CoV-2) have been implicated [19, 20]. Considering the paucity of kinin level data for respiratory lavage fluids and diverging plasma levels, determining endogenous profiles in healthy individuals is indispensable to allow the identification of altered kinin levels in COVID-19 and other diseases.

Therefore, it was aimed to comprehensively study biological levels and variations in kinin peptide profiles (KD, Hyp^4^-KD, KD_1-9_, BK, Hyp^3^-BK, BK_1-8_, BK_1-7_, BK_1-5_, and BK_2-9_) in nasal fluids and plasma within a population of healthy volunteers using the above mentioned LC–MS/MS platforms.

## Materials and methods

### Study design

This study was conducted per the principles expressed in the Declaration of Helsinki and approved by the ethics committee of the medical faculty at the Heinrich Heine University (study number: 6112). All participants provided written informed consent before their enrollment. Bioanalysis was conducted in compliance with Good Clinical Laboratory Practice. Healthy volunteers above the age of 18 years without any signs of respiratory infection or acute allergy were recruited. Volunteers taking drugs interfering with the KKS were excluded. Participants were tested for COVID-19 (Panbio™ COVID-19 Ag Rapid, Abbott Laboratories, IL, USA) to rule out asymptomatic SARS-CoV-2 infection before biological fluid sampling. Venous blood, nasal lavage fluid (NLF) and demographic data were collected from the volunteers.

### Blood sampling

A standardized protocol, proven to significantly limit inter-day and interindividual variability of kinin levels in plasma was employed for the collection of venous blood [16]. Blood was collected in the upright position into 2.7 mL K3 ethylenediamine tetraacetic acid S-Monovettes® (Sarstedt, Nümbrecht, Germany) prespiked with customized protease inhibitor under aspiration [21]. Sampling into three consecutive tubes was performed to confirm adequate blood sampling by assessing inter-tube variability of BK, which was aimed to be < 2 pM. In addition, a fourth tube was drawn in the absence of inhibitors to monitor the impact of lack of inhibitors and inappropriate sampling on the artificial generation of plasma kinin levels. Therefore, protease inhibitor was added to the fourth tube 15 min after venipuncture.

Additionally, it was investigated whether kinin levels were subject to circadian rhythms. Blood was taken from a male and a female subject at the following time-points: 6 a.m., 9 a.m., 12 p.m., 3 p.m. and 6 p.m.

Blood was sampled using 21 G Safety Multifly® needles with 200 mm tubing (Sarstedt, Nümbrecht, Germany) from the left or right median cubital, cephalic, or basilic vein with the needle inserted in an antegrade fashion. Blood samples were immediately centrifuged at 2,000 ×* g* for 10 min at room temperature. Plasma was stored at − 80 °C until analysis.

### Sampling of nasal lavage fluid

Nasal lavage was performed with 10 mL of 0.9% saline (B. Braun, Melsungen, Germany) using 5 mL pre-filled syringes for each nostril and a Schnozzle® Nasal Irrigation Adapter (Splash Medical Devices, LLC, GA, USA). The volunteers were asked to tip their heads backwards, hold the breath, and refrain from swallowing. The fluid obtained was collected directly into the protease inhibitor and was vortexed after completing the sampling. At least 30% of the instilled volume had to be recovered during the lavage in line with the American Thoracic Society guideline for bronchoalveolar lavage [22]. The samples were centrifuged at 4 °C for 15 min at 500 ×* g* to remove cells, mucus, and debris. NLF samples were stored at − 80 °C until analysis.

### Estimating kinin levels in nasal epithelial lining fluid

Kinin levels in NLF are diluted by lavage fluid and therefore do not represent endogenous kinin levels. To allow for the estimation of kinin levels in endogenous nasal epithelial lining fluid (NELF), the dilution of urea in NLF compared to plasma was determined. Therefore, urea nitrogen was measured by an enzyme immunoassay (EIABUN, Invitrogen™, Carlsbad, CA, USA) in NLF (undiluted) and plasma (1:20 dilution) of healthy volunteers. The urea nitrogen assay performance was confirmed to be linear (mean R^2^ = 1 [n = 4]), accurate (within- and between accuracy between − 1.9 and 2.2% at three quality control levels [low, mid, high]) and precise (within-run and between-run precision < 3.6% at three quality control levels [low, mid, high]), applying the customized protease inhibitor.

The dilution factor was then calculated by Eq. 1 according to Kaulbach et al. [23]:

1 $$Dilution\,factor\,(DF)= \frac{[{Urea}_{Plasma}(mg/dL)]}{[{Urea}_{NLF}(mg/dL]}$$

Calculation of the dilution factor by nasal lavage.

Using the determined individual-dependent dilution factor, kinin levels were corrected for the dilution by nasal lavage as follows (Eq. 2 [23]):

2 $${Kinin\,conc.}_{NELF}={Kinin\,conc.}_{NLF}*DF$$

Calculation of the kinin concentration in NELF.

In addition, the volume of sampled NELF by lavage was calculated [23]:

3 $${V}_{NELF\,sampled}=\frac{V_{collected}}{DF}*\frac{V_{lavage}}{V_{collected}-(V_{collected}/DF)}$$

Calculation of the nasal epithelial lining fluid volume sampled.

### Mass spectrometric kinin quantification

The following kinins were quantitatively assessed: kallidin (trifluoracetic acid (TFA) salt, 96.9%, high-performance liquid chromatography (HPLC); Tocris, Bristol, UK), BK (acetate salt, 99.0%, HPLC; Sigma-Aldrich, St. Louis, MO, USA), and their metabolites BK_1-8_ (acetate salt, 98.7%, HPLC; Santa Cruz Biotechnology, Dallas, TX, USA), BK_1-7_ (TFA salt, ≥ 95.0%, HPLC; GenScript, Piscataway Township, NJ, USA), BK_1-5_ (TFA salt, ≥ 95.0%, HPLC; GenScript), BK_2-9_ (TFA salt, ≥ 95.0%, HPLC; GenScript), and KD_1-9_ (TFA salt, 95.9%, HPLC). In addition, hydroxylated BK and KD were determined: Hyp^4^-KD (≥ 99%, HPLC, Peptanova, Sandhausen, Germany) and Hyp^3^-BK (≥ 99%, HPLC, Peptanova). [Phe8Ψ(CH-NH)-Arg9]-BK (TFA salt, 97.5%, HPLC, Tocris) was applied as the internal standard. LC–MS/MS platforms in plasma and respiratory lavage fluids had been successfully validated according to regulatory bioanalytical guidelines of the US Food and Drug Administration [24] regarding precision, accuracy, sensitivity, linearity, matrix effects, recovery and stability. Both platforms are characterized by lower limits of quantification (LLOQ) down to 1.9 pM (depending on the kinin). Details on the assay characteristics have been published elsewhere [13, 14]. All stated peptide concentrations were corrected for salt content and peptide purity, referring to the conducted amino acid analysis.

### Data analysis

LC–MS/MS data acquisition was conducted using Analyst® 1.6.2 software (AB Sciex, Darmstadt, Germany) and raw data evaluation was executed using Multiquant™ 3.0.2 (AB Sciex, Darmstadt, Germany). Statistical analysis and graphics were generated using OriginPro 2021 (9.8.0.200). Descriptive statistics (mean ± standard deviation (SD) or median [interquartile range (IQR)]) and box-whisker-plots were used to describe kinin level data. Outcomes were analyzed using the Mann–Whitney U-test or the two-sided t-test.

## Results

### Study population

In total, 28 volunteers were enrolled. These were white with a median of 26.5 [25–28] years. Of those, 11 were female and 17 were male. Plasma was successfully sampled from 24 subjects and NLF samples with a recovered volume of more than 30% were collected from 24 subjects. COVID-19 antigen tests were negative for all volunteers. Detailed demographics can be found in Table 1.

**Table 1:** Characteristics of the healthy volunteers. Data are expressed as median [interquartile range] or number (n (%))

Demographics	Volunteers		
	All (n = 28)	Male (n = 17)	Female (n = 11)
Age [years]	26.5 [25–28]	27 [25–30]	26 [24–27]
Caucasians (n (%))	28 (100.0)	17 (100.0)	11 (100.0)
Medication (n (%))	7 (25.0)	2 (11.8) - insulin – 1 (5.9) - metoprolol – 1 (5.9)	4 (36.4) - hormonal contraception – 4 (36.4)
Reported allergies (n (%)) (all inactive)	10 (35.7)	8 (47.1) - dust mite – 3 (17.6) - pollen – 4 (23.5) - insect sting – 1 (5.9) - nuts – 1 (5.9)	2 (18.2) - pollen – 1 (9.1) - penicillin – 1 (9.1)

### Endogenous kinin levels in plasma

**Fig. 2:**
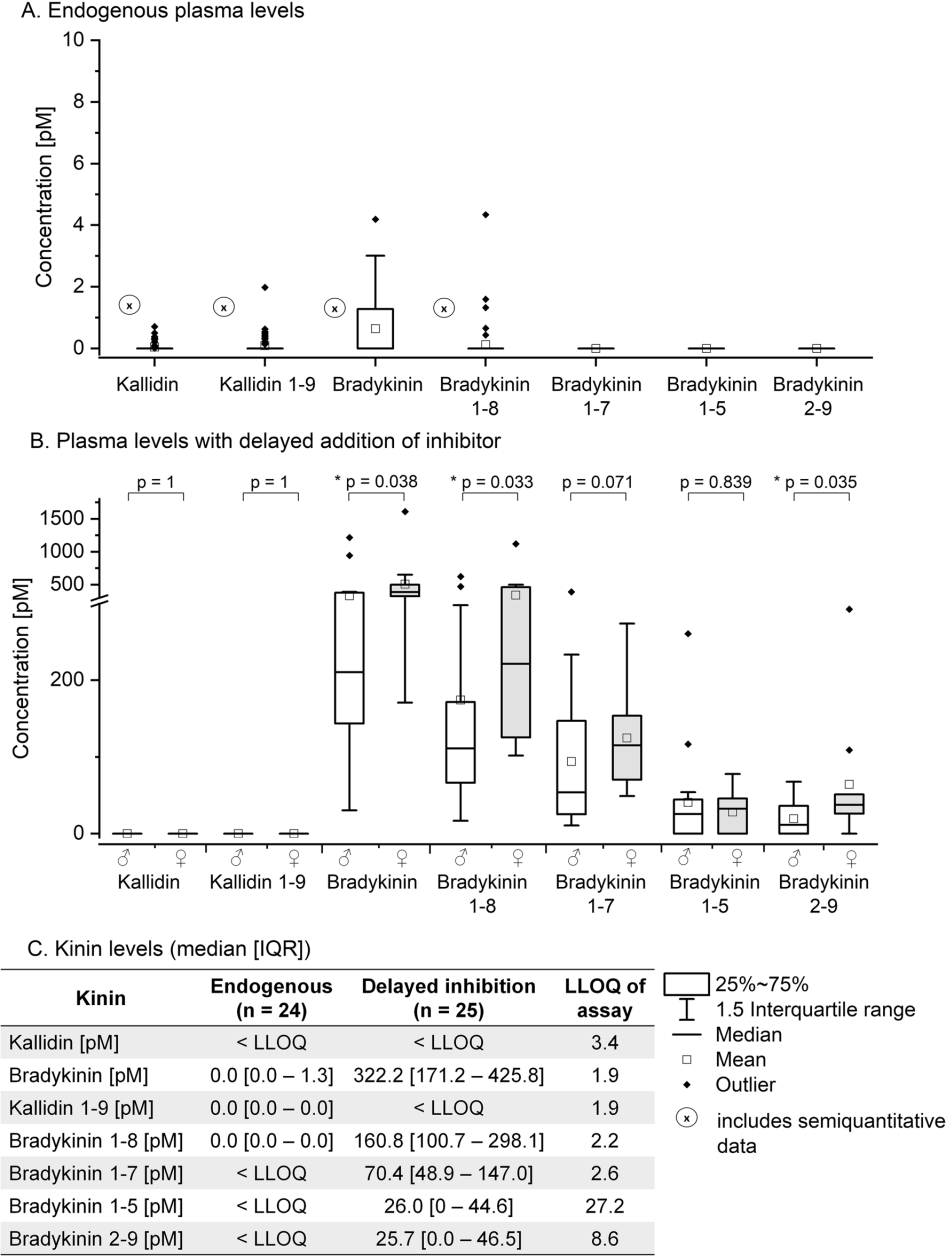
Kinin levels in plasma of healthy individuals. Box plots of endogenous levels are displayed in **A** (n = 24). Box plots of artificially altered kinin levels are presented in **B** (n = 25). An overview of median [interquartile range (IQR)] levels is shown in **C** LLOQ: lower limit of quantification

For 24 subjects, endogenous levels of kinins fell in the very low pM range, with inter-tube variations below 1.7 pM (median 0.0 [− 0.2 to 0.3] pM). Median BK levels were 0.0 [0.0 to 1.3] pM, with maximum level of 4.2 pM. Detected kinin levels below the validated lower limit of quantification (LLOQ) of the LC–MS/MS platform were set as 0. Other kinin levels were still lower and to a large extent below the LLOQ; therefore, values between the detection limit and LLOQ were recorded semi-quantitatively. For des-Arg(10)-kallidin, we measured levels from 0.2 pM (*minimum*) to 2 pM (*maximum*; n = 10), for BK_1-8_ 0.4 pM (*minimum*) to 4.3 pM (*maximum*; n = 5), and for KD 0.2 pM (*minimum*) to 0.7 pM (*maximum*; n = 9) (Fig. 2). No gender-related differences in kinin concentrations were observed.

Furthermore, these low endogenous levels were confirmed throughout the day (6 a.m. to 6 p.m.) in one male and one female subject. No association between circadian rhythms and kinin levels was found.

### Monitoring of kinin profiles applying inappropriate sampling conditions in plasma

Kinin levels in samples artificially altered by delayed addition of inhibitor after 15 min substantially increased compared with endogenous levels for BK (median 322.2 [171.2–425.8] pM) and its metabolites BK_1-8_ (median 160.8 [100.7–298.1] pM), BK_1-7_ (median 70.4 [48.9–147.0] pM), BK_2-9_ (median 25.7 [0.0–46.5] pM), and BK_1-5_ (median 26.0 [0–44.6] pM). KD and KD_1-9_ resulted below their LLOQ. Artificial generation affected BK most, and BK_1-8_ was the main detectable metabolite with a percentage metabolite/BK ratio of 47.6% (median) after 15 min. Lower rates were found for BK_1-7_ (median 17.6%), BK_2-9_ (median 5.7%), and BK_1-5_ (median 3.8%). While no significant differences were observed between men and women regarding the relative formation of BK metabolites, certain absolute kinin levels differed significantly. A more pronounced formation of BK was found in female individuals (female: 410.9 [317.5–570.1] pM, male: 223 [152.2–401.3] pM, *p* = 0.038). While levels of BK_1-5_ and BK_1-7_ did not vary by gender, significant gender-specific differences in the generation of BK metabolites were detected for BK_1-8_ (female: 221.4 [125.2–470.3] pM, male: 111.1 [66.5–171.5] pM, *p* = 0.033) and BK_2-9_ (female: 65.6 [37.5–292.6], male: 36.3 [11.6–67.7] pM, *p* = 0.035).

### Kinins in nasal lavage fluid of healthy volunteers

**Fig. 3:**
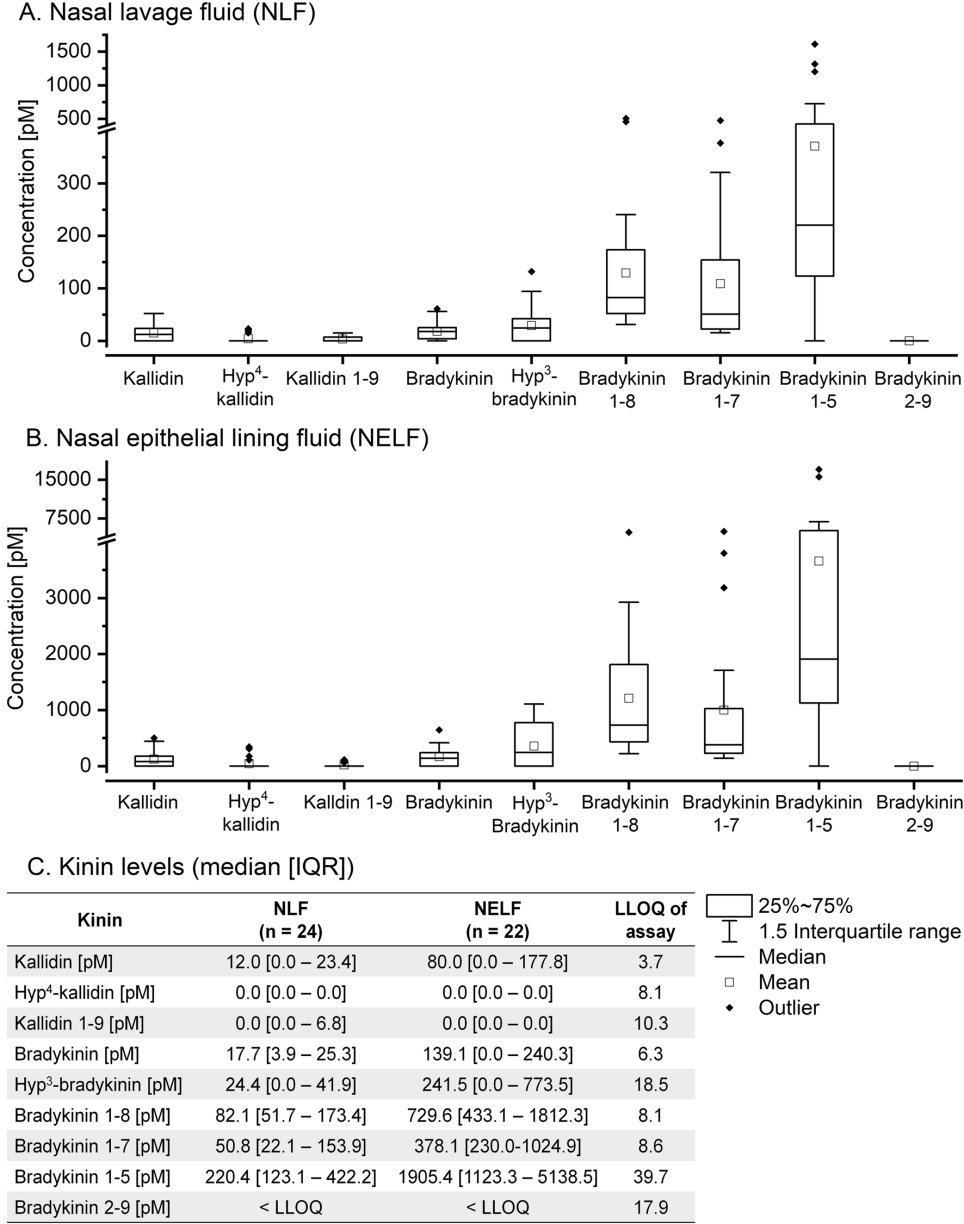
Box plots of kinin levels in nasal lavage fluid (NLF) normalized to the return volume (**A**; n = 24) and nasal epithelial lining fluid (NELF, **B**; n = 22) of healthy volunteers are depicted. An overview of median [interquartile range (IQR)] levels is shown in **C** LLOQ: lower limit of quantification

**Fig. 4:**
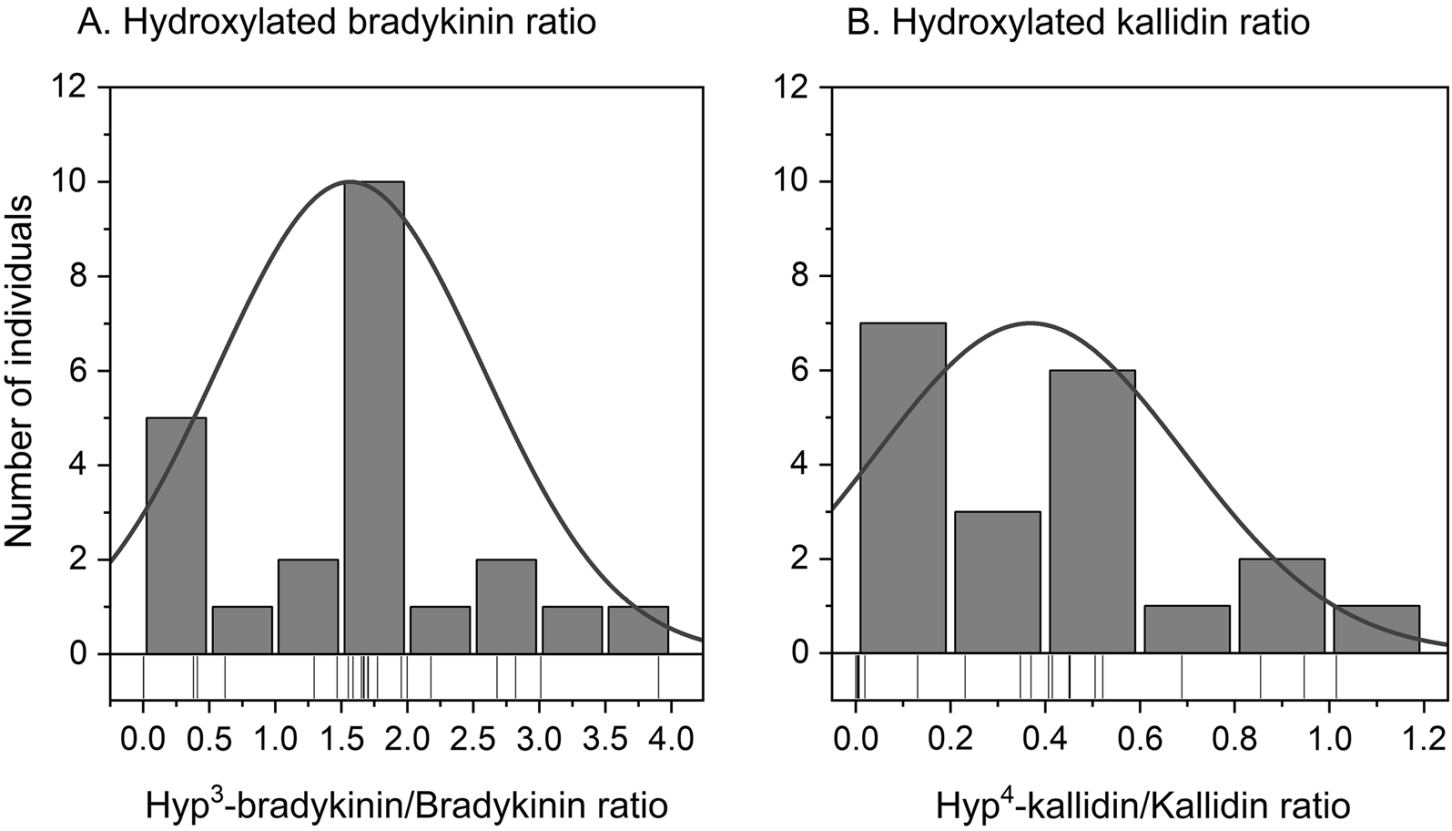
Histogram with normal distribution curve of the hydroxylated kinin to non-hydroxylated kinin ratio for bradykinin (**A**) and kallidin (**B**). Individual data points are shown as thin lines on the x-axis

The mean percentage return volume of nasal lavage with 10 mL of saline was 73.1 ± 11.5% (Table 2). Kinin levels in NLF normalized to the return volumes were low for KD (12.0 [0.0–23.4] pM) and BK (17.7 [3.9–25.3] pM) (Fig. 3). For BK, the hydroxylated form approximated the non-hydroxylated form with a mean ratio of 1.6 ± 1.0 (Fig. 4A). Levels of hydroxylated KD were lower than non-hydroxylated kallidin with a mean ratio of 0.37 ± 0.33 (Fig. 4B). Low levels of the kallidin metabolite des-Arg(10)-kallidin were only detectable in seven volunteers, resulting in median levels of 0.0 [0.0–6.8] pM. Higher concentrations were found for BK_1-8_ (82.1 [51.7–173.4] pM), BK_1-7_ (50.8 [22.1–153.9] pM) and BK_1-5_ (220.4 [123.1–422.2] pM). Levels of BK_2-9_ fell below the limit of detection. The percentage metabolite/BK ratio was 604.6% (median) for BK_1-8_, 568.1% for BK_1-7_, 1,396.3% for BK_1-5_ and 0% for BK_2-9_. No significant gender-specific differences for any kinin assessed were found in NLF.

**Table 2:** Results for nasal lavage fluid sampling. Data are expressed as mean ± standard deviation or median [interquartile range]. NLF: nasal lavage fluid, NELF: nasal epithelial lining fluid

Nasal lavage	Volunteers		
	All (n = 28)	Male (n = 17)	Female (n = 11)
Recovered volume [%]	73.1 ± 11.5	73.8 ± 13.0	72.0 ± 9.6
Plasma urea [mg/dL]	12.3 ± 3.5	13.8 ± 3.3	10.1 ± 2.6
NLF urea [mg/dL]	1.0 ± 0.6	1.1 ± 0.4	0.9 ± 0.7
Dilution factor	12.3 [10.0–19.0]	12.0 [10.4–16.9]	17.0 [10.0–21.6]
Volume NELF sampled [µL]	883.6 [555.6–1,112.0]	910.4 [628.5–1,061.7]	623.5 [484.8–1,112.0]

### Estimating endogenous kinin levels in nasal epithelial lining fluid

The amount of NELF sampled was estimated by correlation of plasma and NLF urea. The median dilution factor in NLF was calculated to be median 12.3 [10.0–19.0] and the determined median NELF volume sampled was median 883.6 [555.6–1,112.0] µL (Table 2). Estimated endogenous kinin levels in NELF were 80.0 [0.0–177.8] pM for KD, 0.0 [0.0–0.0] pM for Hyp^4^-KD, 139.1 [0.0–240.3] pM for BK, 241.5 [0.0–773.5] pM for Hyp^3^-BK, 378.1 [230.0–1,024.9] pM for BK_1-7_, 729.6 [433.1–1,812.3] pM for BK_1-8_, and 1,905.4 [1,123.3–5,138.5] pM for BK_1-5_. KD_1-9_ was quantifiable in five healthy volunteers (80.1 [75.5–109.0] pM, n = 5), but below the LLOQ in most subjects, resulting in median levels of 0.0 [0.0–0.0] pM. Higher levels of KD_1-9_ in these five individuals did not correlate with higher levels of other kinins. NELF levels for BK_2-9_ were not estimated, as these fell below the limit of detection in NLF. The percentage metabolite/BK ratio was median 581.2% for BK_1-8_, 731.2% for BK_1-7_, 1,533.4% for BK_1-5_ and 0% for BK_2-9_. No significant gender-specific differences were found.

### Comparing kinin profiles in plasma and nasal lavage fluid

**Fig. 5:**
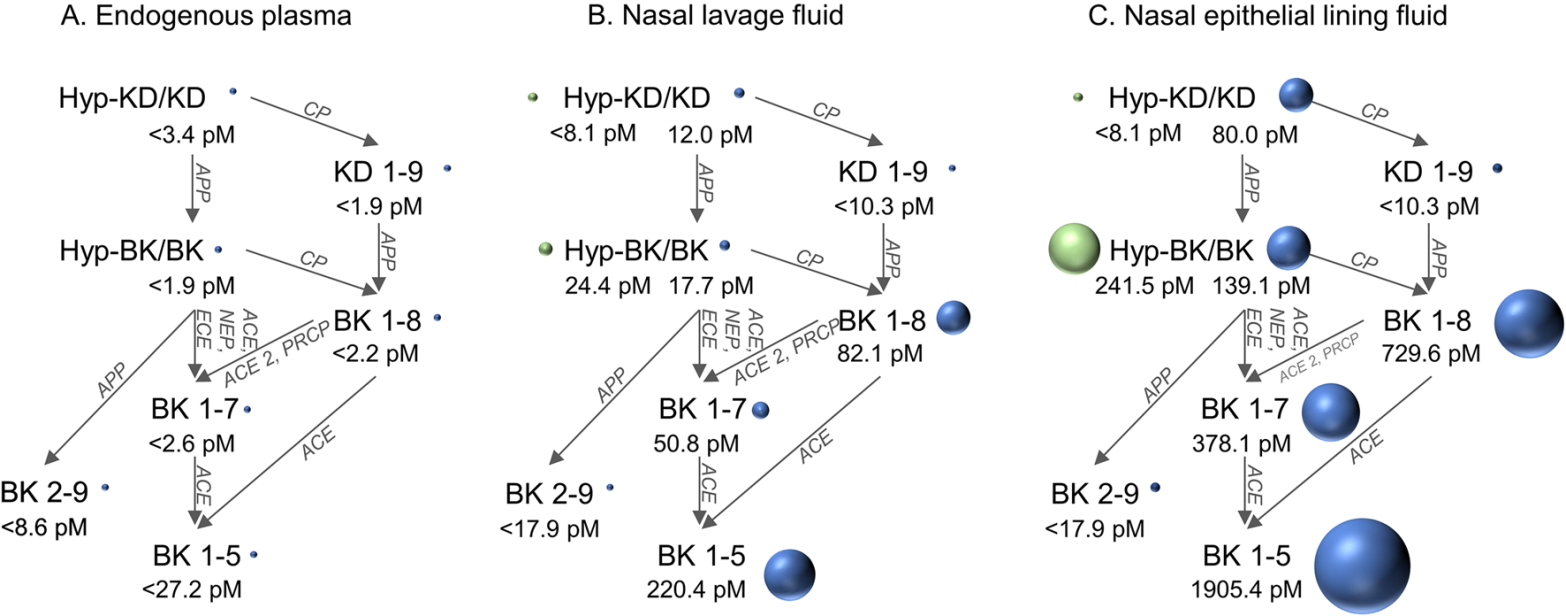
Comparison of endogenous kinin profiles in plasma (**A**), nasal lavage fluid (**B**) and nasal epithelial lining fluid (**C**). The ball size correlates with kinin concentration. ACE: angiotensin-converting enzyme, ACE 2: angiotensin-converting enzyme 2, APP: aminopeptidase P, ECE: endothelin-converting enzyme, BK: bradykinin, CP: carboxypeptidase, Hyp: hydroxylated proline position 3 (BK) or 4 (KD), KD: kallidin, NEP: neprilysin, PRCP: prolyl carboxypeptidase

Endogenous plasma levels of kinins were substantially lower compared to endogenous levels in NELF (Fig. 5). In plasma, only BK levels were detectable, while other metabolites fell below the quantification limit in most volunteers. In NLF and NELF, higher levels of BK compared to plasma were found by a factor of 13.6 (NLF) and 107.0 (NELF). In addition, KD and KD_1-9_ were quantifiable in NLF and NELF, whereas in plasma, these were only rarely detectable in samples. In contrast to plasma, endogenous metabolites of BK were detectable in NLF and NELF, with BK_1-5_ representing the most abundant kinin.

## Discussion

Within this study, kinin profiles in nasal fluids and plasma were assessed in healthy adult volunteers, a population commonly used as control groups in studies exploring biomarkers. This study presented the first comprehensive determination of nine kinin peptides and allowed the compilation of kinin profiles in 24 healthy adult individuals. While endogenous plasma levels were in the very low picomolar range, endogenous NELF levels were in the high picomolar to low nanomolar range. We found no significant gender-specific differences of endogenous kinin levels in plasma, NLF, or NELF.

Plasma kinin levels diverging in several orders of magnitude have been published thus far. For example, while Nussberger et al. measured BK levels of 2.2 pM (n = 22), van den Broek et al. found levels of 100.7 nM (n = 6), a difference by a factor of approximately 50,000 in healthy volunteers [8, 25]. These conflicting data hinder the comparison of data collected in this study with previously published data on kinins. Reliable research on endogenous kinin levels requires stabilization of short-lived kinins with a suitable inhibitor and control of the artificial generation of BK by factor XII-mediated contact activation during sample collection and handling [16, 21]. A standardized protocol was used in this study to measure reliable kinin levels, which allowed to confirm the blood sampling and handling process by evaluating the inter-tube variability (< 1.7 pM). The so collected BK level data in healthy volunteers confirmed low levels of circulating BK in plasma [9, 25–27]. Moreover, comprehensive data for six further BK-related peptides were assessed in plasma and enhanced available data for BK metabolites in plasma [9, 28, 29].

To date, only two studies have published data on kinin levels in NLF. These studies detected immunoreactive BK levels between < 18.9–141.5 pM [30] and median 64.2 pM [31] in eight volunteers, respectively. Assessment of immunoreactive BK does not differentiate BK from KD or their hydroxylated forms. In contrast, within this study kinins were selectively measured in 24 volunteers, whereby the sum of collected BK and KD levels and their hydroxylated forms matched previous determinations of immunoreactive BK [sum of medians: 54.1 pM]. However, NLF concentrations do not represent endogenous levels in NELF. Adjusting for differences in plasma and NLF urea, endogenous human NELF kinin levels were estimated for the first time and these levels were found to be higher than in NLF by a median factor of 12.3 using a lavage volume of 10 mL. Altogether, higher levels of kinins were demonstrated in nasal fluids than in plasma, reflecting that the KKS is primarily a tissue-based system [27].

While no significant gender-specific differences were found in endogenous kinin levels in plasma or NELF, a significant difference was found in artificially altered plasma samples. Here, a more pronounced ex vivo generation was detected in women for BK, BK_1-8_ and BK_2-9_. This might reflect the influence of estrogens on the KKS: (1) estrogens can increase kallikrein and kininogen formation [32], (2) they enhance factor XII concentrations in plasma [33] and (3) they reduce ACE activity [34]. While (1) and (2) may explain increased BK formation, (3) might be causative for increased degradation of BK into BK_1-8_ and BK_2-9_ as an escape pathway (Fig. 1). Additionally, aminopeptidase P activity is higher in women compared to men presumably contributing to the increased ex vivo formation of BK_2-9_ in women [35].

Moreover, this study was not restricted on determining only the immunoreactive kinin fraction, but nine kinins were assessed differentially. This is advantageous for several reasons. First, numerous pathways influence the formation and degradation of kinins in vivo, and disease and pharmacological agents may affect these pathways differently. Such effects can now be comprehensively studied using the collected kinin profiles in the present healthy cohort. Second, in addition to BK, other active kinins, such as KD, BK_1-8_, and KD_1-9_, act on different receptors, which in turn are regulated in a disease-specific manner. Comparison of pathological alterations against physiological kinin profiles may help identify new therapeutic targets. Third, kinins exist in both hydroxylated and non-hydroxylated forms [27, 36]. The kinins BK and KD and their respective hydroxylated forms exhibit similar biological activities but may be altered in a disease-specific manner [37–39]. The collection of kinin profiles in healthy volunteers, who frequently serve as control groups in clinical studies, facilitates the investigation of disease-related alterations in kinin profiles. For example, it was found that hypoxia increases BK hydroxylation via increased activity of prolyl-4-hydroxylase-α1 [40], and diseases such as COVID-19 may induce hydroxylation of kinins. Clinical investigation of this and similar hypotheses is now enabled by evaluating reference kinin profiles in healthy individuals.

With advancing scientific methodology, kinins may evolve into promising biomarkers in the future. First attempts were made by applying the here presented approach also to plasma levels in hereditary angioedema patients [41]. In hereditary angioedema with C1 esterase inhibitor deficiency in remission, a significant increase in BK_2-9_, BK_1-5_ and the sum of eleven kinins was found when compared to a healthy collective. Future works are planned to further elucidate the metabolism and generation of kinins endogenously or exogenously in standardized settings to improve the understanding of the KKS in the development of diseases like hereditary angioedema, allergy, sepsis, epilepsy, stroke, and Alzheimer’s disease [6, 11, 42]. The evaluation of kinin profiles in these pathological processes using physiological profiles as a baseline for comparison may establish a better understanding of the pathophysiology, provide evidence for new therapeutic targets, and improve monitoring of the disease course.

## Conclusion

In this study, comprehensive profiles of endogenous kinin profiles were collected in nasal fluids and plasma of healthy adult volunteers, which may serve as control groups in clinical studies exploring the value of kinins as biomarkers. While circulating plasma kinin levels were below 4.2 pM in our subjects, levels in NELF were in the high picomolar to low nanomolar range, depending on the kinin. We found no gender-specific differences in the fluids studied. The knowledge of comprehensive kinin profiles in healthy volunteers now forms the basis for evaluating disease-specific diagnostic or prognostic information of kinin profile alterations in diseases such as angioedema, sepsis, COVID-19, epilepsy, and Alzheimer’s disease.

## Data Availability

The datasets used and/or analysed during the current study are available from the corresponding author on request.

## Abbreviations

ACE: Angiotensin-converting enzyme
ACE 2: Angiotensin-converting enzyme 2
APP: Aminopeptidase P
B1/2: Bradykinin receptor type 1/2
BK: Bradykinin
CP: Carboxypeptidase
DPP IV: Dipeptidylpeptidase IV
ECE: Endothelin-converting enzyme
HMW: High molecular weight
HPLC: High-performance liquid chromatography
IQR: Interquartile range
KD: Kallidin
KKS: Kallikrein-kinin system
LC–MS/MS: Liquid chromatography coupled to tandem mass spectrometry
LLOQ: Lower limit of quantification
LMW: Low molecular weight
NELF: Nasal epithelial lining fluid
NEP: Neprilysin
NLF: Nasal lavage fluid
PRCP: Prolyl carboxypeptidase
RAAS: Renin–angiotensin–aldosterone system
RNA: Ribonucleic acid
SARS-CoV-2: Severe acute respiratory syndrome coronavirus 2
SD: Standard deviation
TFA: Trifluoroacetic acid
